# A comprehensive ultra-wideband dataset for non-cooperative contextual sensing

**DOI:** 10.1038/s41597-022-01776-7

**Published:** 2022-10-22

**Authors:** Mohammud J. Bocus, Robert Piechocki

**Affiliations:** School of Computer Science, Electrical and Electronic Engineering, and Engineering Maths, University of Bristol, Bristol, BS8 1UB UK

**Keywords:** Computer science, Electrical and electronic engineering

## Abstract

Nowadays, an increasing amount of attention is being devoted towards passive and non-intrusive sensing methods. The prime example is healthcare applications, where on-body sensors are not always an option or in other applications which require the detection and tracking of unauthorized (non-cooperative) targets within a given environment. Therefore, in this paper we present a dataset consisting of measurements obtained from Radio-Frequency (RF) devices. Essentially, the dataset consists of Ultra-Wideband (UWB) data in the form of Channel Impulse Response (CIR), acquired via a Commercial Off-the-Shelf (COTS) UWB equipment. Approximately 1.6 hours of annotated measurements are provided, which are collected in a residential environment. This dataset can be used to passively track a target’s location in an indoor environment. Additionally, it can also be used to advance UWB-based Human Activity Recognition (HAR) since three basic human activities were recorded, namely, sitting, standing and walking. We anticipate that such datasets may be utilized to develop novel algorithms and methodologies for healthcare, smart homes and security applications.

## Abstract (summary)

**Table:** 

Measurement(s)	Human physical activity • Human location
Technology Type(s)	Ultra-wideband impulse radar
Factor Type(s)	Human location • Human physical activity • Monitoring environment • Participant demographics • Radio-frequency signals
Sample Characteristic - Organism	Homo sapiens
Sample Characteristic - Environment	residential building
Sample Characteristic - Location	United Kingdom

## Background & Summary

Over the past few decades, indoor localization has become an active area of research due to the increasing demands for location-based services. Indoor localization is the process of finding the precise location of devices, people or objects in indoor environments, where the Global Positioning System (GPS) service is mostly unreliable. Nowadays, a lot of attention is being directed towards the use of ubiquitous Radio-Frequency (RF) signals for indoor positioning since they overcome the limitations of computer vision-based methods which include high costs of deployment, breach of people’s privacy and limitation to Line-of-Sight (LoS) scenarios^1^. Indoor localization systems can be classified as active and passive based on the target’s participation^2^. In the former, the target carries a device (tag) that actively participates or collaborates in the localization process. Conversely, the main idea behind passive indoor localization is to use existing sensing infrastructure for monitoring the target’s activities and tracking its motion in indoor environments (target does not carry any device). Human (or object) motion has some impact on the sensing environment, and by continuously monitoring variations in the wireless channel, the location of the target can be estimated, usually with a lower accuracy than device-based localization methods. These two types of localization systems meet the requirements of various applications in different ways. For example, in an airport the passengers may want to navigate to their boarding gates and hence their devices, e.g. smartphones, need to actively participate in the localization process. However, in surveillance and security applications, we cannot presume that the targets are carrying an active transmitting device for us to track their location^3^. This may also be the case in healthcare applications where, for instance, the location of elderly people or patients with known disabilities needs to be monitored, without having them to wear uncomfortable body sensors.

Over the past two decades, the coarse-grained Received Signal Strength Indicator (RSSI)^4–9^ and fine-grained Channel State Information (CSI)^10–19^, which can be extracted from a few WiFi Network Interface Cards (NICs), have emerged as dominant methods for both active and passive radio-based indoor localization. Passive localization based on the fingerprinting approach has mostly been considered in these works. The RSSI-based fingerprinting approaches^4–6^ have achieved an average accuracy between 2–5 m. The drawback of RSSI is that it suffers from temporal fluctuations in complex indoor environments because of multipath induced fading, even in a static environment^20^. Furthermore, fingerprinting approaches require a substantial radio-map survey in the offline training phase and labor-intensive and time-consuming fingerprint updates when there are changes in the environment^18^. Additionally, this technique assumes the target is immobile at each position, which may not always be the case in a real-world environment. On the other hand, WiFi CSI has been used in conjunction with algorithms such as Multiple Signal Classification (MUSIC) to estimate the Angle-of-Arrival (AoA) and Time-of-Arrival (ToA) of incoming signals for active localization^16–18^. However, the AoA-based methods usually require the deployment of several WiFi access points equipped with multiple receiving antennas. CSI, which is in frequency domain, can be converted to Channel Impulse Response (CIR) using Inverse Fast Fourier Transform (IFFT). Device-to-device localization using ToA information consists of identifying the direct wave in the CIR profile and once its ToA is known, the range can be calculated by simply multiplying it with the speed of light. The main issue with the ToA method is that it is influenced by the bandwidth and considering the low sampling rate of the 20 MHz and 40 MHz WiFi channel bandwidths (temporal resolutions of 50 ns and 25 ns, respectively), the direct signal may arrive between sampled intervals. The works which have considered ToA for localization usually combine CSI data from several WiFi channels to obtain a larger bandwidth, and thus a better time resolution to disentangle the multiple paths in the channel^17^. However, in practice, we cannot use all channels in both the 2.4 GHz and 5 GHz bands simultaneously so as not to interfere with other WiFi systems and also the wireless regulatory body imposes strict regulations in terms of bandwidth/channel usage. Furthermore, WiFi chipsets suffer from hardware errors which distort the phase information, making accurate localization very challenging, unless strict synchronization (via cables) is implemented between the WiFi transmitter and receiver^21,22^. However, this defeats the purpose of a WiFi system.

Therefore, for applications that require sub-meter level localization accuracy, RF signals other than RSSI and CSI should be employed. In this regard, Ultra-Wideband (UWB) technology incorporating a wide frequency bandwidth (usually in excess of 500 MHz) has been used for providing ranging and positioning with centimetre-level accuracy in many indoor applications. Although UWB technology has been around for a while, it is not until recently that affordable UWB chipsets have been commercialized for civilian applications and this technology is now part of the IEEE 802.15.4 standard. The first companies to commercialize UWB products include Time Domain (now part of 5D Robotics, Inc.,) and Ubisense. More recently, companies such as Bespoon and Decawave (now acquired by Qorvo) have joined the race to provide cheap UWB integrated solutions for ranging and localization. These commercially-available UWB systems are built upon the active localization strategy which relies on the targets (e.g., people or objects) to carry a device (tag), and the ranging is usually performed using Time-Difference-of-Arrival (TDoA), ToA or Two-Way Ranging (TWR) protocol, depending on the UWB chipset manufacturer. These types of systems have been used in applications such as healthcare^23^, robot navigation^24^ and assistant living^25^. However, in some applications such as crime-prevention, surveillance, intruder detection, elderly care, and emergency response, it is impractical to use these active devices^26,27^. Throughout the years, passive multi-static UWB radar systems have been studied for various applications, including detection and localization of people behind obstacles^28–30^, emergency rescue operations^31–33^, target detection and tracking^26,34–47^, object localization and occupancy detection^2,48,49^, health, security and safety^25,50–55^, entertainment and smart home applications^56,57^, people detection through respiratory movement^58,59^ and Human Activity Recognition (HAR)^60,61^. It should be noted that other unobtrusive RF methods such as WiFi CSI and Passive WiFi Radar (PWR) have shown promising results for the HAR task^62–64^. Another technique that is widely used for HAR is based on wearable inertial sensors^65–68^. The most commonly used inertial sensor-based HAR datasets include *UCI-HAR*, *PAMAP2*, *WISDM*, *UNIMIB-SHAR*, and *OPPORTUNITY*. Compared to inertial sensor-based and video-based methods for HAR, RF-based techniques bring the following advantages: (1) they enable device-free/contactless sensing (users do not have to wear uncomfortable body sensors, especially those suffering from skin diseases), (2) they are unobtrusive and privacy-friendly, (3) they ensure a low-cost and ease of deployment, (4) they have wide coverage (we are constantly surrounded by RF signals and thus dead zone is not an issue), and (5) RF-based systems work equally well in the dark and well-lit environments. Regarding the use of radar devices in healthcare applications, a dataset is provided in^69^, which consists of children vital signs measurements recorded with a Frequency-Modulated Continuous Wave (FMCW) radar and a clinical sensor. Open-source UWB datasets have been made available for a number of applications. These include gesture recognition^70^, motion detection/recognition^71–74^, passive target localization (including fingerprinting-based approaches)^36,75^, people counting^76^, and active indoor localization/positioning in LoS and/or NLoS scenarios^77–88^. In a closely related work, a multimodal dataset consisting of WiFi CSI, Passive WiFi Radar (PWR), UWB and Kinect measurements, is proposed for tasks like passive target localization and HAR^89^. In this work, we bring the following contributions:

- There are currently very few open-source datasets on passive localization and some of these are either limited in data length, environmental layout, and/or consider only one particular application. Furthermore, current commercial pulse-based UWB systems are mainly used for device-to-device communication and active localization. Therefore, in this work, we extend the capability of such systems from active localization to non-cooperative sensing. Namely, we propose a more comprehensive dataset that can be concurrently used for the passive (uncooperative) localization of a moving target as well as for the recognition of basic human activities.
- The experiment is performed in a residential environment spanning over several rooms. Our choice of monitoring environment relies on the emergence of new Internet-of-Things (IoT) applications such as smart home health/activity monitoring and person-tracking. Our dataset can help in developing new techniques or algorithms that can be used, for example, in healthcare facilities/homes to monitor patients’ behaviours or activity level (without the use of wearable sensors or intrusive video cameras), and in so-doing medical institutions can measure their patients’ health status remotely and in real-time. Approximately 1.6 hours of RF measurements are fully annotated with location and activity labels. The participant’s motion and natural behavior are captured, as would be the case in a real-world scenario. The dataset is comprehensive since it contains approximately 3 Million annotated data points.

This publicly available dataset is meant for both non-collaborative localization and HAR, which are areas of growing interest to research communities focusing on wireless sensing, radar and Internet of Things (IoT) technologies. To ensure that the dataset aligns to the FAIR (Findable, Accessible, Interoperable, Reusable) Data principles of Open Science, we have (i) made it open-access via the share repository, (ii) provided a detailed description of the dataset, (iii) formatted our dataset using standard filetypes and encoding, and (iv) provided an example script that will allow the user to load and analyze the data.

## Methods

**Fig. 1:**
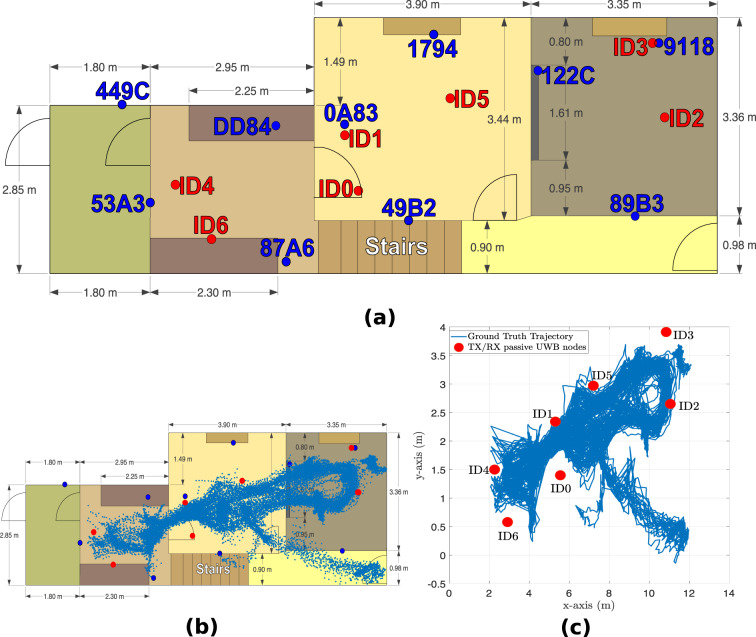
Experimental layout: (**a**) floor plan; (**b**) footprint of activities and (**c**) ground truth target trajectory.

**Fig. 2:**
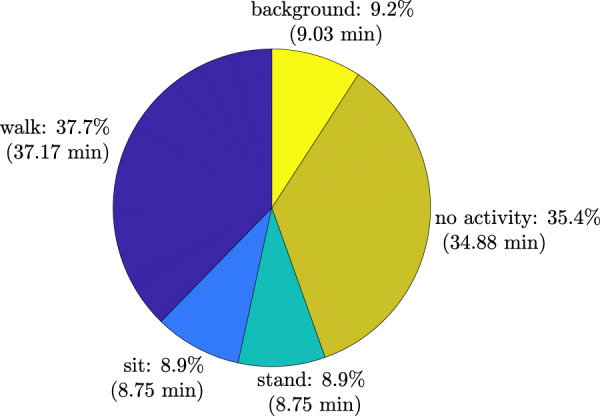
Experiment activities distribution.

The experiment was performed in an actual residential environment across four furnished rooms (including corridor) on the ground floor level, with dining table, chairs, TV screen, sofa, fridge, and other objects commonly found in a residential setting lying in the surroundings (high clutter environment). The experimental layout is depicted in Fig. 1 along with the footprint of activities and ground truth target trajectory. The target performed three main activities, namely, walking, sitting and standing for an approximate experiment duration of 1.6 hours, including steady state (no activity, target not moving). The breakdown of the activities’ durations is given in Fig. 2. The background data refers to the data collected in the monitoring area when the target was not present. Two UWB systems were used during the experiment. The first system (see blue nodes in Fig. 1(a)) was used to obtain the ground truth position of the target while he/she carried a tag and moved within the monitoring area. Ten fixed anchor nodes were used for this purpose.

The passive UWB system (red nodes in Fig. 1(a)) was used to capture CIR data from the signals reflected off the target while he/she performed the different activities. It consisted of seven fixed Qorvo’s EVK1000^90^ modules installed in a multi-static configuration and operating as transceivers. The modules were programmed with a custom firmware so as to record CIR data on all modules. The CIR samples (each being a complex number) reported by the Qorvo’s UWB chipset have a sampling interval of Δ*τ*_s_≈1.0016 ns^91^, which means that the best range resolution that can be achieved using the CIR data is ~30 cm. Only 50 CIR samples out of 1016 (for the Pulse Repetition Frequency of 64 MHz) were captured in each measurement across the nodes in the multi-static UWB system. This corresponds to a sensing range of 15 m for each node. Each CIR measurement was extracted from the accumulator memory of the chipset, starting 3 samples (3 ns) before the detected first path index as reported by the Leading Edge Detection (LDE) algorithm in the chipset. The detected first path index is reported in the FP_INDEX field in register 0 × 15 of the DW1000 chipset^91^. Node ID0 was configured as the initiator (INIT) to initiate the exchange of Single-Sided Two-Way Ranging (SS-TWR) messages (*poll*, *response* and *final*)^92^ with each of the other 6 modules. When a node transmits a frame, the latter is heard by all other nodes operating on the same channel. Hence, each node can read the received frames in their accumulator and extract the CIR data. This means that each node acts as a transceiver, resulting in 21 bidirectional communication links. All 7 nodes were connected to laptops to log the CIR data using a serial terminal. The average sampling rate was around 24 Hz between each transmit-receive pair (bidirectional) and therefore considering all 21 bidirectional links, the sampling rate of the passive UWB system amounted to approximately 504 Hz. The corresponding ground truth positions of the nodes deployed in the two systems are given in Tables 1,2. The other parameters used in the two UWB systems are specified in Table 3.

**Table 1:** Coordinates of ground truth UWB nodes (blue). Bottom left corner of floor plan is taken as origin.

Active UWB node ID	X (m)	Y (m)	Z (distance above ground in m)
1794 (INIT)	6.90	4.05	1.83
89B3	10.52	0.98	1.62
9118	10.95	3.91	1.17
122 C	8.78	3.44	1.75
0A83	5.30	2.53	1.73
449 C	1.30	2.85	1.75
53A3	1.80	1.20	1.80
87A6	4.25	0.20	1.70
DD84	4.05	2.50	1.75
49B2	6.45	0.90	2.07

**Table 2:** Coordinates of passive UWB nodes (red).

Passive UWB node ID	X (m)	Y (m)	Z (distance above ground in m)
ID0 (INIT)	5.55	1.40	0.70
ID1	5.30	2.34	1.73
ID2	11.05	2.65	0.84
ID3	10.84	3.91	1.16
ID4	2.26	1.50	0.89
ID5	7.20	2.97	0.85
ID6	2.91	0.58	0.95

**Table 3:** Ground truth and passive UWB systems’ parameters (*maximum receiver bandwidth is approximately 900 MHz).

Parameter	Ground truth UWB system (blue)	Passive UWB system (red)
Channel number	5	4
Carrier frequency	6489.6 MHz	3993.6 MHz
Bandwith	499.2 MHz	1331.2* MHz
Pulse repetition frequency	64 MHz	64 MHz
Data rate	6.8 Mbps	6.8 Mbps
Preamble length	128 symbols	128 symbols
Preamble acquisition chunk size	8	8
Preamble code	9	17

Throughout the experiment, no personal data was recorded from the participant. Nevertheless, the participant was made fully aware of the purpose of carrying out the experiment and his/her role in it. Informed consent was obtained from the participant prior to the experiment. All studies that fall under the OPERA - Opportunistic Passive Radar for Non-Cooperative Contextual Sensing project were thoroughly reviewed and fully approved by the University of Bristol Faculty of Engineering Research Ethics Committee (application number: 96648). Risk assessment was also performed and approved prior to the experiment.

### Ground Truthing

- The Qorvo’s MDEK1001 equipment^93^ was utilized to get the ground truth location of the target within the monitoring area. Ten units were configured as anchors and mounted on walls across the rooms in the residential environment (see blue nodes in Fig. 1(a)). Their mounting positions (see Table 1) were measured with the help of a laser measuring device, which were then entered in the Qorvo’s DRTLS Android app^94^. Two additional UWB units, each connected to a laptop, were also configured as listeners for the sole purpose of recording the ground truth *xy* coordinates of the tag, along with their timestamps with millisecond precision. The ground truth *xy* coordinates reported by the tag were logged at an update rate of 10 Hz on each UWB listener.
- Video ground truth was used for the purpose of labelling the activity data. This means that during the experiment, a video camera featuring a millisecond timestamp functionality, was deployed to continuously record the various activities i.e., sitting, standing, walking and no activity. The passive UWB nodes (red nodes in Fig. 1(a)) were connected to laptops, and they recorded the CIR data along with their millisecond timestamps. All recording devices were appropriately synchronized to the same local Network Time Protocol (NTP) server. Therefore, the video recording, CIR data from the passive UWB system and ground truth *xy* coordinates obtained from the active UWB system, were all synchronized using their millisecond timestamps when curating the dataset. Activity labels were extracted manually, that is, by visualizing the video recording of the experiment and analysing which activity was performed at a given point in time. Then the activity labels were synchronized with the CIR data and ground truth *xy* coordinates of the target using their recorded timestamps.

## Data Records

The UWB dataset, which is intended for passive localization and HAR can be accessed and downloaded from our figshare repository^95^. The different files available in our curated dataset are specified in Table 4. The dataset files are provided in both .mat format and the more common .csv format to ensure that users can open the files using software such as MATLAB or Python. The dataset files contain complex CIR data, timestamps, details about the transmitting and receiving nodes, activity labels, ground truth *xy* coordinates of the target, as well as some other useful UWB parameters that are reported by the Qorvo’s DW1000 chipset. The file background_CIR basically contains the CIR data collected in the environment using the multi-static UWB system (red nodes in Fig. 1(a)) when no target was present. This was done at the start of the experiment. The file target_CIR contains the CIR data recorded when the target was performing various activities such as walking, sitting and standing (including the no activity portions where the target was at rest).

**Table 4:** Dataset file details.

Filename	No. of samples	File formats
target_CIR	2,670,072	.mat and.csv
background_CIR	269,295	.mat and.csv

### UWB dataset description

This section describes the structure of the data files specified in Table 4. The dataset files are available in .csv and MATLAB .matformats and each row in the files corresponds to a received UWB packet. The columns in the dataset have the following headers:

- timestamp (column 1): Timestamp in milliseconds when the UWB packet was received.
- CIR1,CIR2,…, CIR50 (columns 2–51): These correspond to the 50 complex CIR samples logged in each received packet between a given transmitter-receiver pair.
- FP_index (column 52): This is the accumulator first path index as reported by the Leading Edge Detection (LDE) algorithm of the DW1000 UWB chipset in register 0 × 15 (in FP_INDEX field). It is a sub-nanosecond quantity, consisting of an integer part and a fractional part.
- FP_Amp1 (column 53): First path amplitude (index 3) value reported in the FP_AMPL1 field of register 0 × 15 of the DW1000 chipset.
- FP_Amp2 (column 54): First path amplitude (index 2) value reported in the FP_AMPL2 field of register 0 × 12 of the DW1000 chipset.
- FP_Amp3 (column 55): First path amplitude (index 1) value reported in the FP_AMPL3 field of register 0 × 12 of the DW1000 chipset.

It should be noted that FP_Amp1, FP_Amp2 and FP_Amp3 represent the magnitudes of the accumulator tap at the indices 3, 2 and 1, respectively, beyond the integer part of FP_INDEX reported in register 0 × 15 of the DW1000 chipset^91^.

- maxGrowthCIR (column 56): Channel Impulse Response (CIR) power value reported in the CIR_PWR field of register 0 × 12 of the DW1000 chipset. This value represents the sum of the squares of the magnitudes of the accumulator from the estimated highest power portion of the channel, which is related to the receive signal power^91^.
- rxPreamCount (column 57): Preamble Accumulation Count (PAC) value reported in the RXPACC field of register 0 × 10 of the DW1000 chipset. RXPACC reports the number of accumulated preamble symbols. The DW1000 chip estimates the CIR by correlating a known preamble sequence with the received signal and accumulating the result over a time interval^96^. The number of preambles used for estimating the CIR depends on the received signal quality^97^. And since the magnitudes of the CIR depend on the number of preamble symbols, the magnitude values have been normalized using the PAC value for each CIR measurement in the dataset^37^.
- maxNoise (column 58): Maximum value of noise as reported by the DW1000 chipset^98^.
- stdNoise (column 59): Standard deviation of Channel Impulse Response Estimate (CIRE) noise value as reported in the STD_NOISE field of register file 0 × 12 of the DW1000 chipset^91^.
- Estimated_dist_between_Init_ID0_and_other_nodes_metres (column 60): This is the distance (in metres) estimated by the initiator (node ID0) when it performs Two-Way Ranging (TWR) with each of the other 6 nodes.
- True_dist_between_Tx_Rx_metres (column 61): This is the true distance (in metres) measured between each pair of nodes.
- FP_pow_dbm (column 62): This is the estimated first path power level (in dBm) of the UWB signal between a pair of nodes.
- rx_pow_dbm (column 63): This is the estimated received power level (in dBm) of the UWB signal between a pair of nodes.

The formulas for calculating the FP_pow_dbm and rx_pow_dbm values are provided in^91^. According to Qorvo, these two parameters can help to deduce whether the received signal is LoS or Non-Line-of-Sight (NLoS). It is stated that, as a rule of thumb, if the difference between the two parameters, i.e., rx_pow_dbm - FP_pow_dbm is less than 6 dB, the signal is most likely to be LoS, while a difference greater than 10 dB means that the signal is most probably NLoS^91^.

- Tx_ID (column 64): ID of the transmitting node. The possible transmitting node IDs are 0, 1, 2, 3, 4, 5 or 6 (see red nodes in Fig. 1(a)).
- Rx_ID (column 65): ID of the receiving node. The possible receiving node IDs are 0, 1, 2, 3, 4, 5 or 6 (see red nodes in Fig. 1(a)).
- Tx_Pos_X,Tx_Pos_Y (columns 66–67): *x*- and *y*- coordinates of the transmitting node, respectively.
- Rx_Pos_X,Rx_Pos_Y (columns 68–69): *x*- and *y*- coordinates of the receiving node, respectively.
- GT_X_coord, GT_Y_coord (columns 70–71): This is the ground truth position of the target in the monitoring area in terms of its 2D *x*- and *y*- coordinates.
- Activity (column 72): Refers to the current activity being performed, specified as a string of characters e.g., “walk”, “sit”, “stand”, “bignoactivity” and “noactivity”. The activity label “noactivity” refers to the case where the person was not performing any activity, that is, his/her body was at rest, for example between activities such as “sitting” and “standing”. “bignoactivity” refers to a prolonged amount of time when the user was standing still and was not performing any activity. This was recorded prior to each walking activity segment. These portions of data can periodically be used to update or re-calibrate the background CIR with the dynamic CIR data (for example using background subtraction methods) when tracking the moving target passively. The “no activity” and “bignoactivity” CIR data portions can also be used for motion detection purposes, with a binary output of “1” indicating motion detected and “0” otherwise. Additionally, they can also be used in conjunction with the background CIR data to detect the location of the target even if the latter is immobile in the environment. This is because the presence of the target in the monitoring environment will impact the CIR multipath profile when compared to the background CIR data.

Columns 70–72 are not present in the background_CIR.mat file since no target was present during background data recording.

## Technical Validation

**Fig. 3:**
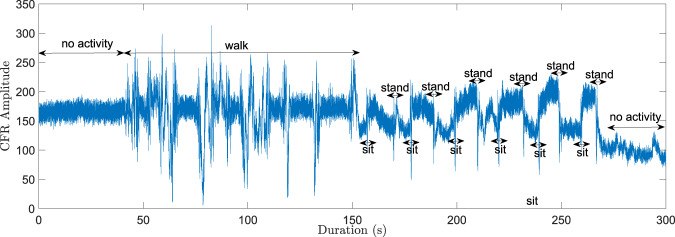
Signal analysis of UWB CFR data for a 300-second time window (considering the 10^th^ CFR sample between node ID0 and node ID2 for the passive UWB system).

**Fig. 4:**
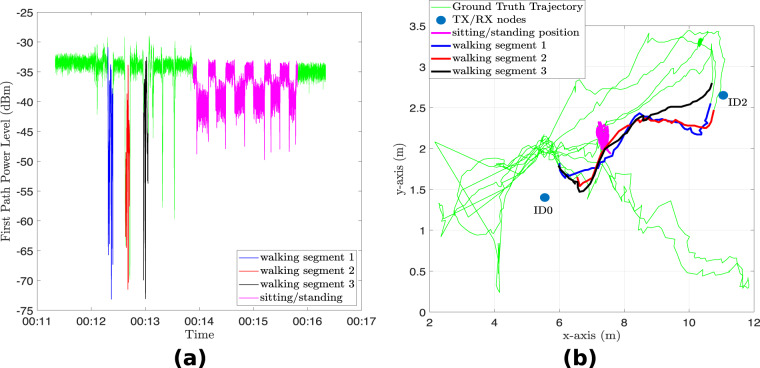
(**a**) Estimated first path power level (dBm) between node ID0 and node ID2 for a time window of 300 seconds and (**b**) corresponding target trajectory.

**Fig. 5:**
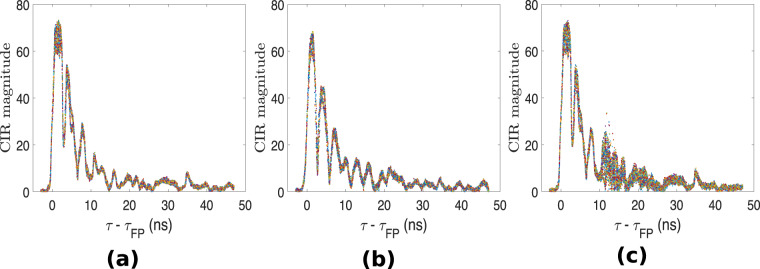
500 accumulated and aligned CIR measurements recorded between nodes ID0 and ID4 of passive UWB system: (**a**) empty room; (**b**) target standing still and (**c**) target performing sitting and standing activities. Note: *τ*_FP_ represents the first path (direct) signal time-of-flight between the pair of nodes.

Figure 3 shows the signal analysis between node ID0 and node ID2 in the multi-static UWB system, considering a 300-second duration time window. The raw CIR data has been converted to Channel Frequency Response (CFR) using the Fast Fourier Transform (FFT) and the signal is plotted for the 10^th^ CFR sample. The various activities cause different patterns in the signal and these signal patterns can serve as input to machine/deep learning algorithms to recognize human activities. The raw CIR can also be utilized for the same purpose, as demonstrated in^60^ where a high activity recognition performance was achieved. Figure 4(a) shows the first path power level recorded between node ID0 and node ID2 for the same time window and the corresponding trajectory is shown in Fig. 4(b). As can be observed in Fig. 4(a), there are significant drops in the first path power level at certain time instants between the pair of devices. At these points in time, the target was located somewhere between the two devices, obstructing the LoS signal, as illustrated in Fig. 4(b) for a few cases. In this 300-second time window, the sitting and standing activities were performed near the LoS path between the two nodes. The resulting pattern in the first path power level for these two activities is similar to the one obtained in Fig. 3 using the CFR data. If there are significant drops in first path power level between two or more pairs of nodes and they coincide at a given point in time, we can infer the position of the target at this particular instant. Therefore, the first path power level can be used in conjunction with the CIR data in a multi-static device configuration to achieve passive target localization. Figure 5 shows 500 accumulated and aligned CIR measurements recorded between a given pair of UWB modules under different conditions. It can be observed that the received signal consists of the direct path pulse followed by signal reflections due to both the target and clutter. As can be observed from Fig. 5(a,b), when the room is empty or the target is still (no motion), the accumulated CIR measurements are stable. However, when the target is in motion, variations occur in the CIR, as can be observed in the region *τ*-*τ*_FP_ ≈12 ns in Fig. 5(c). The earliest point in time at which these variations occur in the CIR is often referred to as the bi-static delay^99^. The transmitting/receiving nodes are fixed in the multi-static network and their positions are known. Therefore, the distance covered by the direct (first path) signal between pairs of nodes can be calculated along with its delay *τ*_FP_. It should be noted that the bidirectional CIR data exchanged between a given pair of devices is reciprocal, i.e., the CIRs in the forward direction and backward direction should be identical according to antenna reciprocity principle^37^. Provided that the signal emitted from the transmitter reflects off the target and reaches the receiver without further scattering, then the bi-static range defines an ellipse showing the possible locations of the target^36,100^. The transmitter and receiver are the foci points on this ellipse and the length of its major axis is equivalent to the bi-static range. Ideally, the common intersection point of all ellipses due to multiple transmitter and receiver pairs indicates the exact location of the target. The interested reader may refer to the works in^3,35,36^ for further discussions on the concept of how to extend the functionality of pulse-based UWB systems from active localization to passive localization. In our previous work^3^, we use a succession of well-defined signal processing steps to passively track a person with good localization accuracy using only the reported CIR data. We leverage the variations in the target CIR data (refer to Fig. 5) that stand out against the background CIR data to find the Time-of-Flight (ToF) of the signal (caused by moving target) between each transmitter-receiver link and combine them, for example using Taylor series or intersection of ellipses method, along with Kalman tracking to find the 2D coordinates of the moving target.

It should be noted that the UWB modules in the multi-static configuration have independent RF clocks and thus the CIR measurements exchanged between pairs of modules may be sampled at different times^36^. The first path index (FP_INDEX) value for each CIR measurement is reported as a real number (usually around 750^101^) having a resolution of $\frac{1.0016{\rm{ns}}}{64}$ (see column FP_index in dataset files). Since each CIR measurement typically has a different first path index value, the CIR measurements which are accumulated over a number of samples in time need to be aligned with their respective estimated FP_INDEX, and the latter can be shifted to be at the start of the CIR buffer, as depicted in Fig. 5. That is, the time axis is shifted to be zero at the rising edge for each individual CIR measurement^75^. A MATLAB script is included with this dataset and it includes an example on how to accumulate and align the recorded CIR measurements. Moreover, as stated in^36^, those CIR measurements where the number of accumulated preamble symbols (see column rxPreamCount in UWB datasets) is less than half of the number of transmitted preamble symbols (128 in this case) are regarded as outliers and can thus be dropped.

### HAR performance using UWB CIR data

**Fig. 6:**
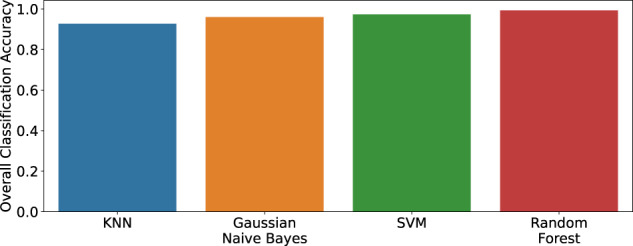
HAR classification accuracy using different machine learning algorithms (considering CIR data between nodes ID0 and ID2 only).

**Fig. 7:**
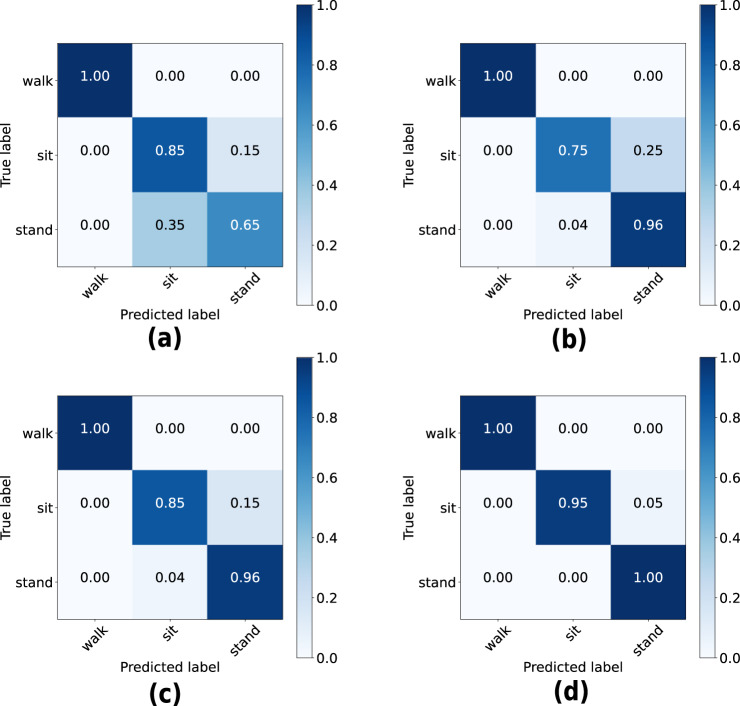
Confusion matrices depicting the HAR performance using different machine learning algorithms: (**a**) KNN; (**b**) GNB; (**c**) SVM and (**d**) Random Forest.

In this section we evaluate the HAR performance using the UWB CIR as features, to assess the technical quality of the dataset and demonstrate its potential usefulness for the activity recognition task and in so-doing provide a baseline. For this purpose, we considered four basic machine learning algorithms, namely, Gaussian Naive Bayes (GNB), K-Nearest Neighbors (KNN), Random Forest and Support Vector Machine (SVM) to classify the three activities: “walk”, “sit” and “stand”. For the KNN algorithm, the number of neighbors was set to 5. A maximum depth of 50 was selected for the Random Forest algorithm while a linear kernel was considered in the SVM algorithm. 80% of the dataset was used for training while the remaining 20% was used for testing. The same random seed is used for the train/test split to ensure fair comparison when comparing the performance of the different algorithms. In this evaluation, only the bidirectional CIR data between node ID0 and node ID2 is considered. However, the CIR data between all 21 bidirectional links may be regarded as multi-view data when a given activity is being performed at a particular instant in time. The users are encouraged to test different multi-view fusion methods on this dataset by considering the data across all pairs of nodes. Figure 6 shows the overall classification performance of the four algorithms when UWB CIR data are used as features. As can be observed, all four machine learning algorithms achieve an accuracy above 90% for the classification of the three activities. The Random Forest algorithm achieves the highest accuracy (99.34%), followed by SVM (97.37%), GNB (96.05%) and KNN (92.76%). The corresponding confusion matrices are also shown in Fig. 7.

## Usage Notes

The user is encouraged to use the example script provided with the dataset to load and analyze the data. The functionalities of the script are described in the following section.

## Data Availability

A MATLAB script has been made available in the dataset directory for the users to replicate some of the figures in this Data Descriptor: • plot_uwb_signals.m: This script can be used to load the two files background_CIR and target_CIR (either in .csv format or .mat format) and plot the Channel Frequency Response (CFR) of the UWB data for a desired time segment (between a chosen pair of UWB nodes), as illustrated in Fig. 3. Furthermore, this script allows the users to plot the first path power level (in dBm) and ground truth trajectory, as depicted in Fig. 4, and the aligned CIR measurements as demonstrated in Fig. 5.
